# A Novel Multivariate Approach to Phenotyping and Association Mapping of Multi-Locus Gametophytic Self-Incompatibility Reveals *S, Z*, and Other Loci in a Perennial Ryegrass (Poaceae) Population

**DOI:** 10.3389/fpls.2017.01331

**Published:** 2017-08-02

**Authors:** Daniel Thorogood, Steven Yates, Chloé Manzanares, Leif Skot, Matthew Hegarty, Tina Blackmore, Susanne Barth, Bruno Studer

**Affiliations:** ^1^Institute of Biological, Environmental and Rural Sciences, Aberystwyth University Aberystwyth, United Kingdom; ^2^Molecular Plant Breeding, Institute of Agricultural Sciences, ETH Zurich Zurich, Switzerland; ^3^Teagasc Crops Environment and Land Use Programme, Oak Park Research Centre Carlow, Ireland

**Keywords:** DUF247, gametophytic, genome wide association studies (GWAS), principal components analysis (PCA), pollen-stigma incompatibility, *S*-locus, self-incompatibility (SI), *Z*-locus

## Abstract

Self-incompatibility (SI) is a mechanism that many flowering plants employ to prevent fertilisation by self- and self-like pollen ensuring heterozygosity and hybrid vigour. Although a number of single locus mechanisms have been characterised in detail, no multi-locus systems have been fully elucidated. Historically, examples of the genetic analysis of multi-locus SI, to make analysis tractable, are either made on the progeny of bi-parental crosses, where the number of alleles at each locus is restricted, or on crosses prepared in such a way that only one of the SI loci segregates. Perennial ryegrass (*Lolium perenne* L.) possesses a well-documented two locus (*S* and *Z*) gametophytic incompatibility system. A more universal, realistic proof of principle study was conducted in a perennial ryegrass population in which allelic and non-allelic diversity was not artificially restricted. A complex pattern of pollinations from a diallel cross was revealed which could not possibly be interpreted easily *per se*, even with an already established genetic model. Instead, pollination scores were distilled into principal component scores described as Compatibility Components (CC1-CC3). These were then subjected to a conventional genome-wide association analysis. CC1 associated with markers on linkage groups (LGs) 1, 2, 3, and 6, CC2 exclusively with markers in a genomic region on LG 2, and CC3 with markers on LG 1. BLAST alignment with the Brachypodium physical map revealed highly significantly associated markers with peak associations with genes adjacent and four genes away from the chromosomal locations of candidate SI genes, *S-* and *Z-DUF247*, respectively. Further significant associations were found in a *Brachypodium distachyon* chromosome 3 region, having shared synteny with *Lolium* LG 1, suggesting further SI loci linked to *S* or extensive micro-re-arrangement of the genome between *B. distachyon* and *L. perenne*. Significant associations with gene sequences aligning with marker sequences on *Lolium* LGs 3 and 6 were also identified. We therefore demonstrate the power of a novel association genetics approach to identify the genes controlling multi-locus gametophytic SI systems and to identify novel loci potentially involved in already established SI systems.

## Introduction

Many flowering plants possess self-incompatibility (SI) mechanisms that prevent inbreeding by blocking fertilisation of ovules by self or self-like pollen. These mechanisms have evolved independently several times and a number of different plant family-specific systems have been described (Franklin-Tong, 2008). It is notable that all of the systems that have been functionally characterised are under single locus control. Though considerably more challenging, a number of historical attempts have been made to describe multi-locus SI systems, the most thorough being those of Arne Lundqvist for the gametophytic systems of a range of species, but most notably Poaceae species (Lundqvist, 1956), *Ranunculus* spp. (Lundqvist et al., 1973; Lundqvist, 1990) and *Beta vulgaris* L. (Lundqvist et al., 1973). The SI system of the grass family is the multi-locus system where some advances have been made toward gene identification and characterisation. The SI system is recognised as being controlled gametophytically by two complementary loci, *S* and *Z*, which both consist of large polyallelic series. However, even though the mode of action of SI in grasses was reported 60 years ago (Hayman, 1956; Lundqvist, 1956) and confirmed in perennial ryegrass (*Lolium perenne* L.) (Cornish et al., 1979), the identity of the genes has remained elusive despite efforts to map and clone them (Voylokov et al., 1998; Thorogood et al., 2002; Bian et al., 2004; Hackauf and Wehling, 2005; Kakeda et al., 2008; Shinozuka et al., 2010). Very recently, a fine-mapping approach combined with pollen- and stigma-specific gene expression analyses and comparison of sequence diversity of the co-segregating genes from plants of known *S* genotype led to the conclusion that a DUF247 protein acts as the pollen component of the *S* locus on linkage group (LG) 1 (Manzanares et al., 2016). For the *Z* locus, the most convincing candidates are another DUF247 gene found in perennial ryegrass (Shinozuka et al., 2010) and a neighbouring Ubiquitin-Specific Protease (USP) gene found in rye (*Secale cereale* L.) (Hackauf and Wehling, 2005) located on LG 2. In addition, unlinked self-fertility loci have been identified located on LG 5 (Fuong et al., 1993; Thorogood et al., 2005) and LGs 3 and 6 (Wehling et al., 1995). Intriguingly, Thorogood et al. (2002) described a locus on LG 3 that acted epistatically where pollen-specific *S* allele—LG 3 marker allele combinations from a cross between two unrelated plants were not transmitted to their progeny.

The gametophytically controlled reaction of the pollen at the stigma surface enables quantification of the degree of incompatibility between two plants simply by observation of the proportion of compatible and incompatible pollen grains alighting on a stigma surface in so-called semi-*in-vivo* pollinations. Semi-*in-vivo* pollination tests have been used successfully for genetic linkage mapping of SI and self-fertility loci in perennial ryegrass (Thorogood et al., 2002, 2005; Arias Aguirre et al., 2013). Classically, genetic linkage mapping in outcrossing species is based on segregating populations derived from bi-parental crosses between parents with contrasting phenotypes. For SI loci in grasses, these methods have been used, most thoroughly and recently by Manzanares et al. (2016). In this study, the *S* locus was located as the region of maximum marker segregation distortion in families derived from half-compatible crosses. These methods however are restricted to evaluation of single loci, segregating for a maximum of four alleles, and they are dependent on time-consuming preparation and testing of appropriate segregating plant material. In contrast, genome wide association studies (GWAS) on multiple-parent populations of plants do not require preliminary preparation and, as long as population structure is accounted for, are likely to reveal more allelic diversity at several loci simultaneously than that expected from a single bi-parental cross (Kopecký and Studer, 2014). This has the potential to produce a more generalist and robust model and also allows for the prediction of multiple allelic forms of SI loci useful for subsequent marker-based population studies and multiple allelic prediction. Using GWAS in diverse populations also has the potential to reveal novel SI loci that have not been accounted for by existing models. The mapping accuracy is dependent on the marker density and by virtue of several rounds of historical recombination over several generations of within population sexual reproduction (Huang and Han, 2014). Such GWAS approaches to gene discovery were pioneered by human geneticists investigating the genetic control of complex diseases (Purcell et al., 2007) but have been shown to be effective for studying genetic variation in plant populations by identifying associations with *a posteriori* candidate genes (Atwell et al., 2010).

The most systematic approach to describing incompatibility relationships between plants of a multiple-parent population is a full diallel of semi-*in-vivo* pollinations between plants and the evaluation of the proportion of incompatible and compatible pollen grains in each cross. However, the results of the diallel are not immediately amenable to genetic association, because of their multi-dimensional structure: that is each individual genotype is pollinated with itself and, as near as possible, every other genotype within the population. Each pollination score is therefore dependent not only on the genotype itself but also on the genotype of the plant that it is crossed with. Therefore, the pollination diallel builds a complex pattern of the inter-relationships of the plant population. To resolve this complexity of phenotype, Principal Components Analysis (PCA) has been used to partition the multi-dimensional data into few uncorrelated single dimension variables or principal components (Ringnér, 2008) for which individual variates produce relative numerical values. Effectively, where values for plants are similar for a particular component, they are similar in terms of their pollination patterns within the population with regards to that component. Less similar component scores would indicate different pollination behaviour. Similar applications converting raw phenotypic data into PCs subsequently used in genetic association analyses have been reported. For example, PCA was used to identify quantitative trait loci responsible for canid skeleton traits (Chase et al., 2002) and, more recently, to identify how proximal chromatin state influences gene expression and causal chromatin quantitative trait loci (cQTL) (Waszak et al., 2015).

Here, we report a method for quantifying the intra-population incompatibility relationships of a multiple-parent perennial ryegrass population, relating this status to known candidate genes at specific SI loci. The methodology attempts to provide proof of principle evidence that it is possible to identify and locate multiple SI loci of gametophytic systems, even when there is no prior knowledge of either the number of loci segregating or the patterns of segregation expected. A positive outcome would demonstrate the validity of such an approach to understanding the genetic basis of previously undetermined or disputed multi-locus gametophytic SI systems.

## Results

### Pollinations

**Figure 1:**
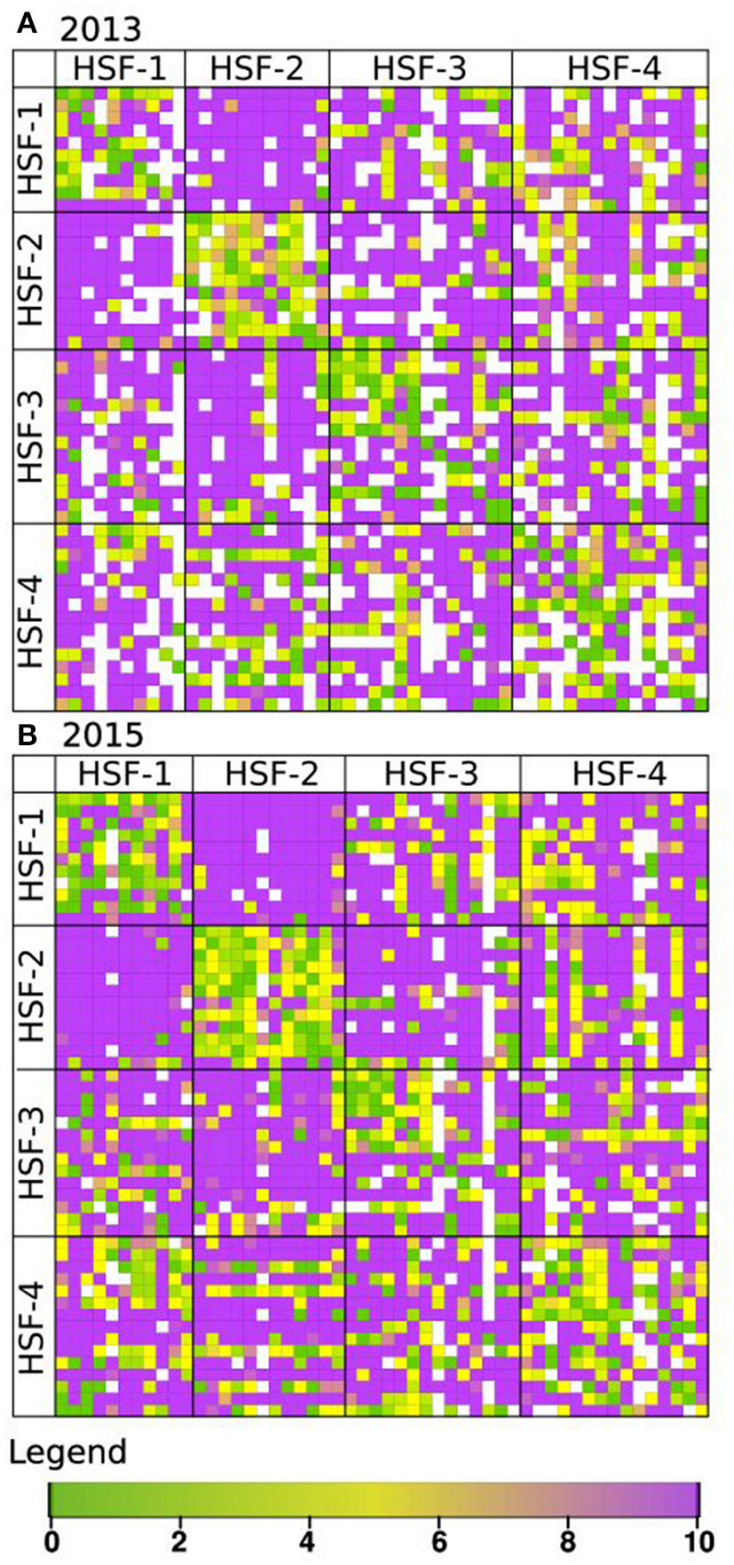
Heat map showing compatibility scores of diallel crosses in the F13 population, evaluated in 2013 **(A)** and 2015 **(B)**. Each grid point represents a single pair-cross between genotypes acting as pollen donor (♂, horizontal) and as pollen recipient (♀, vertical). The four half-sib families (HSF-1 to HSF-4) of the F13 population are indicated by the grid. Compatibility scores range from fully compatible (10, purple) to fully incompatible (0, green). Missing values are represented in white.

Phenotypic characterisation of the perennial ryegrass population, consisting of 52 plants from four half-sib families, was achieved through evaluation of the proportion of pollen tubes germinating in a near-complete diallel cross of all individual plants. Pollinations were made in 2013 and 2015. The results of the semi*-in-vivo* pollinations are represented as heat maps in Figure 1. The number of pollinations successfully completed in 2015 was 2,496 compared to 1,971 in 2013. Two additional genotypes were added in 2015. Nevertheless, the pollination matrices gave similar results overall (*R* = 0.68). Correlations between 2013 and 2015 pollinations for sub-groups of plants were calculated as follows: all plants within each half-sib family; all plants within each half-sib family when crossed with other half-sib families (Supplementary Table 1) and each individual plant's set of pollinations as either the male or the female parent with every other plant (Supplementary Table 2). All of the score sets for within- and between-family comparisons were positively correlated at *P* < 0.001 (Supplementary Table 1). Of the 100 score sets made for crosses between individual genotypes as the male or female parent with other plants in the population, the majority were positively correlated at *P* > 0.001. However, six (as female) and eight (as male) were only correlated at a lower significance level and two sets (genotype 323 used as female parent and 334 as male parent) were not significantly correlated (Supplementary Table 2). Year differences were recorded with, in some extreme cases, fully compatible crosses in 1 year being recorded as fully incompatible in the other.

The pollinations of the diallel cross showed distinctive patterns of half-sib family relationships. The within- and between-family cross-pollination scores were characterised using the SI_50_ population index, which is the quantile at which 50% of the compatibility reactions are half compatible (Supplementary Figures 1, 2). Within-family scores ranged from 0.37 to 0.57 and 0.38 to 0.51 for 2013 and 2015, respectively, compared to between-family scores of 0.03–0.31 and 0.04–0.30 for 2013 and 2015, respectively. Strikingly, nearly all progeny crosses between members of half-sib families 1 and 2 (SI_50_ < 0.032–0.056) were fully compatible (Figure 1), resulting in an average compatibility score of 9.57 and 9.59 in 2013 and 2015, respectively. The results of modelling were found to explain a high proportion of the variation (*R*^2^ > 0.94) and are shown in Supplementary Figures 1, 2 for 2013 and 2015, respectively.

Two features of the pollination scores obtained, in particular, should be noted as they contradict expectations based on a two-locus gametophytic incompatibility system. Firstly, a degree of self-compatibility was observed, with genotype 238, being the most extreme estimated as being 50% self-compatible in both years. Secondly, when a score difference between reciprocal crosses was greater than three it was considered to represent a significantly different compatibility score: a number of pollinations showed reciprocal differences where in one direction the cross was fully compatible. In 2013 and 2015, 174 and 137 of crosses with this conformation, respectively, were identified but in only 10 crosses was this conformation the same in both years.

### Principal components analysis

**Figure 2:**
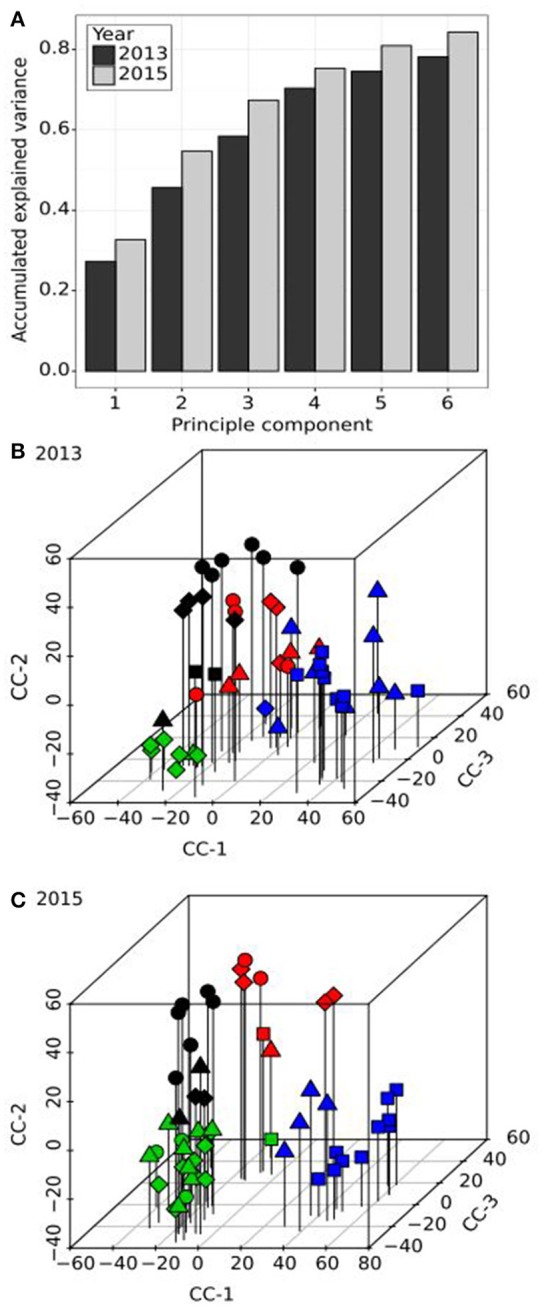
Principal component **(A)** and cluster analysis **(B,C)** of the compatibility scores from diallel crosses in the F13 population. **(A)** The accumulated explained variance (y-axis) of the first six principal components (x-axis) are shown as dark and light grey bars for the evaluations in 2013 and 2015, respectively. **(B,C)** three-dimensional scatterplots of the resulting compatibility components (CC-1 to CC-3) for both years. The shape of the symbols represents the half-sib family origin of each genotype of the F13 population (square, circle, triangle and diamond for half-sib families HSF-1 to HSF-4, respectively). The symbol colours are representative of the first four clusters based on hierarchical clustering analysis (black, red, green, and blue, respectively).

In order to simplify the apparent highly complex pattern of variation in pollination behaviour observed within the population, PCA, applied to the compatibility scores, was employed. The first four principal components (PC) accounted for an accumulated 70% (27, 46, 58, and 70%) of the total variation in 2013 and an accumulated 75% (32, 54, 67, and 75%) of the total variation in 2015 (Figure 2A). The first two PCs correlated well between years (*r*^2^ = 73% and 75%), however the third and fourth PCs appeared to have switched, as PC3 in 2013 correlated better with PC4 rather than PC3 in 2015 (*R*^2^ = 35% as opposed to 9%) and PC4 in 2013 with PC3 rather than PC4 in 2015 (*R*^2^ = 45% as opposed to <1%). The switch of PC3 and PC4 in 2013 and 2015, respectively, might be explained by the proportion of variance attributable to these PCs. In 2013, PC4 accounted for 12% of the variance and likewise in 2015, PC3 accounted for 13% of the variance. In contrast, PC3 in 2013 accounted for 13% but in 2015, PC4 accounted for 8%. The lower percentage accounted for by PC4 in 2015 may be due to the relatively higher proportion of variance explained by PC1 and PC2 (55%) compared to 2013 (46%), which in turn may be due to the higher number of cross-pollinations made. Thus, PCA analysis identified reproducible latent factors in the form of PCs, but their order was not conserved. For this reason, the variation in incompatibility relationships was described as Compatibility Components (CC1-CC3). In 2013, these components equate to PCs 1, 2, and 4, and in 2015 to PCs 1-3. Clear population structures of plant compatibility relationships can be visualised (Figures 2B,C). Although individual genotypes within half-sib families (represented by the different symbols) tended to group together, some individuals were more similar to genotypes from other families. The PC values discriminated four distinct clusters designated by different symbol colours. Representing the data as a dendrogram (Supplementary Figure 3), the four clusters were clearly distinguished based on hierarchical clustering. Individuals from different half-sib families were interspersed within each cluster. 2013 and 2015 clusters, although similar, were not identical.

### Genome wide association analysis

**Figure 3:**
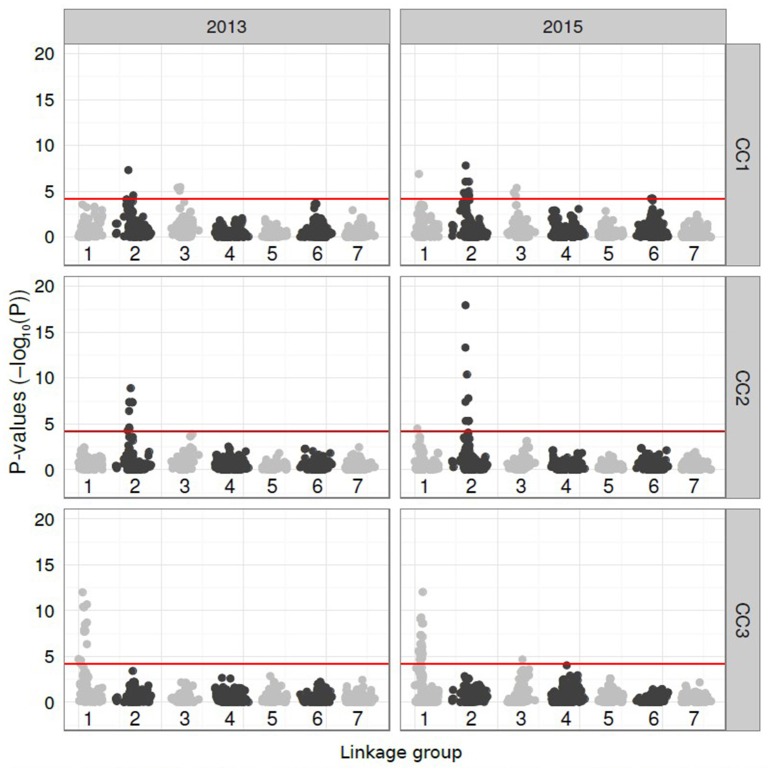
Manhattan plots showing the *P*-values (minus log_10_-transformed, y-axis) of SNP markers located on the seven linkage groups (LGs, x-axis) of perennial ryegrass (*Lolium perenne* L.) for genetic association with the compatibility components (CC-1 to CC-3) calculated from the 2013 and 2015 pollination data. The significance threshold (*P* > 4.233) is shown as a horizontal red line on each plot. SNP markers on odd and even LGs are given in light and dark grey, respectively.

The subsequent GWAS of the CC scores for both years revealed highly significant and consistent associations with markers. All probability scores were plotted against the marker map positions across the seven perennial ryegrass LGs (Figure 3). CC1 revealed significant markers on LG 1 (2015), LG 2 (both years) and LG 3 (both years). For LG 6, markers equalled the Bonferroni correction threshold in 2015 and fell just below in 2013. CC2 revealed highly significant markers exclusively on LG 2 and CC3 on LG 1.

All markers that equalled or exceeded the Bonferroni correction threshold of SI 50 index are listed in Table 1. This listing includes markers that were unmapped but could be attributed to a LG based on their BLAST alignment to the Brachypodium genome sequence and the shared synteny relationship of this sequence to the perennial ryegrass genetic linkage map established by Pfeifer et al. (2013). Also included in Table 1 is a small number of markers with significant association with CC scores where the recombination mapping LG attribution conflicted with the *in silico* mapping prediction. Two markers that were unmapped and could not be attributed to a LG by *in silico* comparative mapping are also included. By far the most frequent and most significant associations were found on LGs 1 and 2.

**Table 1:** Complete list of significantly associated SNP markers, linkage group (LG), map position (cM), -log*P* value, associated compatibility component (CC), year in which association was recorded, predicted *B. distachyon* gene and physical position.

**Marker**	**LG**	**cM**	**−logP**	**CC**	**Year**	**Brachypodium gene**	**Physical position**
**LINKAGE GROUP 1**, **SYNTENIC WITH** ***B. DISTACHYON*** **CHROMOSOME 2**							
Contig7422_2494	1	28.1	7.15	CC3	2015	Bradi2g25745.1	2:23906158-23921663
Contig41583_778	1	26.0	8.61	CC3	2015	Bradi2g25870.1	2:24078750-24081563
Contig41583_192	1	29.5	8.61	CC3	2015	Bradi2g25870.1	2:24078750-24081563
Contig49734_583	U (1)	−	7.33	CC3	2013	Bradi2g31810.1	2:31749059-31753822
Contig49734_583	U (1)	−	4.26	CC1	2015	Bradi2g31810.1	2:31749059-31753822
Contig42828_168	1	21.1	4.31	CC3	2015	Bradi2g31830.1	2:31771118-31781072
Contig41099_600	1	19.2	10.35	CC3	2013	Bradi2g32600.2	2:32517190-32521113
Contig41099_600	1	19.2	6.36	CC3	2015	Bradi2g32600.2	2:32517190-32521113
Contig37988_1146	1	18.3	4.64	CC3	2015	Bradi2g34490.1	2:34557077-34560477
Contig6800_583	U (1)	−	11.99	CC3	2013	Bradi2g35050.3	2:35185369-35190175
Contig6800_583	U (1)	−	6.91	CC1	2015	Bradi2g35050.3	2:35185369-35190175
Contig42271_467	1	14.5	11.99	CC3	2013	Bradi2g35050.4	2:35185369-35190175
Contig42271_467	1	14.5	6.91	CC1	2015	Bradi2g35050.4	2:35185369-35190175
Contig41031_317	U (1)	−	9.23	CC3	2013	Bradi2g35180.1	2:35291194-35296712
Contig41031_317	U (1)	−	4.24	CC3	2015	Bradi2g35180.1	2:35291194-35296712
Contig32050_681	U (1)	−	9.23	CC3	2013	Bradi2g35740.1	2:36164784-36172871
Contig32050_681	U (1)	−	4.24	CC3	2015	Bradi2g35740.1	2:36164784-36172871
**S-DUF247**		−				**Bradi2g35750**	**2:36184898-36187615**
Contig7558_988	U (1)	−	4.72	CC3	2015	Bradi2g36130.1	2:36495631-36505982
Contig35923_413	1	5.2	4.26	CC3	2013	Bradi2g38470.3	2:38769060-38772518
Contig7185_1727	U (1)	−	5.52	CC3	2013	Bradi2g38490.1	2:38776210-38782296
Contig40661_72	1	18.4	5.19	CC3	2015	Bradi2g38590.1	2:38866764-38870433
Contig50074_714	1	0.0	4.72	CC3	2013	Bradi2g39230.1	2:39350812-39356374
Contig34464_527	U (1)	−	6.24	CC1	2015	Bradi2g61830.1	2:58209279-58216738
Contig34464_527	U (1)	−	5.76	CC1	2013	Bradi2g61830.1	2:58209279-58216738
Contig49750_636	U (1)	−	6.03	CC3	2015	Bradi2g62550.1	2:58209279-58216738
**LINKAGE GROUP 1**, **SYNTENIC WITH** ***B. DISTACHYON*** **CHROMOSOME 3**							
Contig50706_721	1	8.5	4.55	CC3	2013	Bradi3g20110	3:19153780-19155999
Contig50706_721	1	8.5	4.49	CC2	2015	Bradi3g20110	3:19153780-19155999
Contig50341_348	1	20.0	9.12	CC3	2015	Bradi3g22760.1	3:21985845-22000222
Contig50341_348	1	20.0	7.72	CC3	2013	Bradi3g22760.1	3:21985845-22000222
Contig41361_697	1	20.0	5.58	CC3	2015	Bradi3g23140.2	3:22451312-22455063
Contig41361_451	1	20.0	4.80	CC3	2015	Bradi3g23140.2	3:22451312-22455063
Contig32184_1849	1	20.9	4.81	CC3	2015	Bradi3g23160.1	3:22516238-22521416
Contig32184_1788	1	26.0	4.81	CC3	2015	Bradi3g23160.1	3:22516238-22521416
Contig51819_286	U (1)	−	5.58	CC3	2015	Bradi3g23240.2	3:22693367-22695291
Contig34497_620	1	20.3	4.80	CC3	2015	Bradi3g24210.1	3:22693367-22695291
Contig34497_956	1	26.0	5.58	CC3	2015	Bradi3g24210.1	3:22693367-22695291
Contig9399_704	U (1)	−	4.80	CC3	2015	Bradi3g26880.1	3:27659755-27661727
Contig7451_450	U (1)	−	5.27	CC3	2015	Bradi3g27877.1	3:29075121-29083057
Contig49757_111	U (1)	−	5.27	CC3	2015	Bradi3g27920.2	3:29155199-29159967
Contig51969_109	1	20.6	5.27	CC3	2015	Bradi3g28220.1	3:29558603-29560390
Contig31470_1993	1	20.7	5.27	CC3	2015	Bradi3g28350.1	3:29680780-29684720
Contig31470_88	1	20.7	5.27	CC3	2015	Bradi3g28350.1	3:29680780-29684720
Contig12252_359	1	23.9	5.27	CC3	2015	Bradi3g28430.1	3:29777174-29779277
Contig12252_491	1	24.7	5.27	CC3	2015	Bradi3g28430.1	3:29777174-29779277
Contig45746_246	U (1)	−	5.27	CC3	2015	Bradi3g28430.1	3:29777174-29779277
Contig9597_1252	1	26.3	5.27	CC3	2015	Bradi3g28460.1	3:29794680-29799909
Contig9597_165	1	26.3	5.27	CC3	2015	Bradi3g28460.1	3:29794680-29799909
Contig9597_606	1	26.3	5.27	CC3	2015	Bradi3g28460.1	3:29794680-29799909
Contig9597_738	1	26.3	5.27	CC3	2015	Bradi3g28460.1	3:29794680-29799909
Contig31167_1317	1	21.2	7.89	CC3	2013	Bradi3g29440.1	3:31508441-31519465
Contig31167_1416	1	21.2	7.89	CC3	2013	Bradi3g29440.1	3:31508441-31519465
Contig31167_2625	1	21.2	7.89	CC3	2013	Bradi3g29440.1	3:31508441-31519465
Contig31167_1317	1	21.2	7.33	CC3	2015	Bradi3g29440.1	3:31508441-31519465
Contig31167_1416	1	21.2	7.33	CC3	2015	Bradi3g29440.1	3:31508441-31519465
Contig31167_2625	1	21.2	7.33	CC3	2015	Bradi3g29440.1	3:31508441-31519465
Contig31167_864	1	21.3	7.89	CC3	2013	Bradi3g29440.1	3:31508441-31519465
Contig31167_864	1	21.3	7.33	CC3	2015	Bradi3g29440.1	3:31508441-31519465
Contig11306_207	1	29.8	8.71	CC3	2013	Bradi3g29970.1	3:32007871-32010510
Contig6880_169	U (1)	−	11.99	CC3	2015	Bradi3g30080.1	3:32089136-32093171
Contig9012_623	1	29.9	10.68	CC3	2013	Bradi3g30430.1	3:32563574-32572371
Contig6965_1844	1	28.7	12.04	CC3	2015	Bradi3g30670.3	3:32868096-32872338
Contig7723_139	U (1)	−	14.81	CC3	2013	Bradi3g30810.1	3:33046837-33051120
Contig18219_251	U (1)	−	9.26	CC3	2015	Bradi3g31460.1	3:33607580-33612312
Contig18219_251	U (1)	−	8.49	CC3	2013	Bradi3g31460.1	3:33607580-33612312
Contig31564_981	1	22.0	9.12	CC3	2015	Bradi3g32000.1	3:34209148-34213422
Contig31564_981	1	22.0	7.72	CC3	2013	Bradi3g32000.1	3:34209148-34213422
Contig31564_549	1	22.4	9.26	CC3	2015	Bradi3g32000.1	3:34209148-34213422
Contig31564_549	1	22.4	8.49	CC3	2013	Bradi3g32000.1	3:34209148-34213422
Contig17281_110	1	25.8	6.05	CC3	2015	Bradi3g33110.1	3:35490833-35495236
Contig17281_168	1	25.8	6.05	CC3	2015	Bradi3g33110.1	3:35490833-35495236
**LINKAGE GROUP 2**, **SYNTENIC WITH** ***B. DISTACHYON*** **CHROMOSOME 5**							
Contig9643_270	U (2)	−	4.40	CC2	2015	Bradi5g15950.1	5:19387633-19388544
Contig31128_1173	2	62.7	4.57	CC1	2013	Bradi5g20012.1	5:22925557-22928526
Contig50239_181	U (2)	−	6.29	CC1	2015	Bradi5g20650.1	5:23462434-23465002
Contig50239_181	U (2)	−	4.23	CC1	2013	Bradi5g20650.1	5:23462434-23465002
Contig6797_1542	2	58.4	7.79	CC2	2015	Bradi5g20940.1	5:23719386-23723013
Contig31275_1341	2	60.1	5.02	CC1	2015	Bradi5g22000.1	5:24485125-24487946
Contig11852_382	U (2)	−	4.27	CC1	2015	Bradi5g23210.1	5:25235365-25236212
Contig32137_513	2	47.8	7.40	CC2	2015	Bradi5g23510.2	5:25420708-25424856
Contig32137_1347	2	47.8	6.06	CC1	2015	Bradi5g23510.2	5:25420708-25424856
Contig32137_513	2	47.8	4.61	CC2	2013	Bradi5g23510.2	5:25420708-25424856
Contig41047_159	2	60.7	6.06	CC1	2015	Bradi5g23660.1	5:25525947-25527067
Contig31123_5169	2	49.0	7.82	CC1	2015	Bradi5g23890.1	5:25670495-25682203
Contig31123_3046	2	49.0	7.35	CC2	2013	Bradi5g23890.1	5:25670495-25682203
Contig31123_3046	2	49.0	5.33	CC2	2015	Bradi5g23890.1	5:25670495-25682203
Contig31123_3046	2	49.0	4.53	CC1	2015	Bradi5g23890.1	5:25670495-25682203
Contig31123_5169	2	49.0	4.49	CC2	2013	Bradi5g23890.1	5:25670495-25682203
Contig31123_1987	2	59.4	7.35	CC2	2013	Bradi5g23890.1	5:25670495-25682203
Contig31123_2674	2	59.4	7.35	CC2	2013	Bradi5g23890.1	5:25670495-25682203
Contig31123_2764	2	59.4	7.35	CC2	2013	Bradi5g23890.1	5:25670495-25682203
Contig31123_1987	2	59.4	5.33	CC2	2015	Bradi5g23890.1	5:25670495-25682203
Contig31123_2674	2	59.4	5.33	CC2	2015	Bradi5g23890.1	5:25670495-25682203
Contig31123_2764	2	59.4	5.33	CC2	2015	Bradi5g23890.1	5:25670495-25682203
Contig31123_1987	2	59.4	4.53	CC1	2015	Bradi5g23890.1	5:25670495-25682203
Contig31123_2674	2	59.4	4.53	CC1	2015	Bradi5g23890.1	5:25670495-25682203
Contig31123_2764	2	59.4	4.53	CC1	2015	Bradi5g23890.1	5:25670495-25682203
**Z-DUF247**		−				**Bradi5g23930**	**5:25719103-25720254**
Contig36905_912	2	53.8	10.39	CC2	2015	Bradi5g24040.1	5:25792245-25793654
Contig36905_912	2	53.8	8.90	CC2	2013	Bradi5g24040.1	5:25792245-25793654
Contig49823_437	2	42.6	4.85	CC1	2015	Bradi5g24220.1	5:25929295-25932276
Contig49823_437	2	42.6	4.29	CC2	2013	Bradi5g24220.1	5:25929295-25932276
Contig6478_1256	2	44.8	7.33	CC1	2013	Bradi5g24370.1	5:26024930-26032253
**OTHER LINKAGE GROUPS**							
Contig33352_272	3	53.6	4.67	CC3	2015	Bradi2g43650.1	2:44108581-44119792
Contig32047_1499	3	29.8	5.07	CC1	2013	Bradi2g61010.1	2:58209279-58216738
Contig32047_1499	3	29.8	4.49	CC1	2015	Bradi2g61010.1	2:58209279-58216738
Contig36358_326	3	32.5	5.47	CC1	2013	Bradi2g61010.1	2:58209279-58216738
Contig36358_326	3	32.5	5.38	CC1	2015	Bradi2g61010.1	2:58209279-58216738
Contig33330_2359	3 (6)	22.7	5.40	CC1	2013	Bradi3g60790.2	3:59608824-59622548
Contig33330_2359	3 (6)	22.7	4.87	CC1	2015	Bradi3g60790.2	3:59608824-59622548
Contig42041_592	6	47.9	4.21	CC1	2015	Bradi3g52437.2	3:53332557-53341325
Contig40572_338	6	46.5	4.21	CC1	2015	Bradi3g52900.3	3:53712002-53718230
Contig17169_307	1 (7)	24.4	7.78	CC3	2013	Bradi1g54210.1	1:52595215-52599078
Contig17169_307	1 (7)	24.4	6.38	CC3	2015	Bradi1g54210.1	1:52595215-52599078
Contig42024_319	4	51.0	4.59	CC3	2015	Bradi1g60320.2	1:59632633-59638818
Contig40660_160	U (7)	−	4.81	CC3	2015	Bradi1g74916.1	1:71954350-71958951
Contig40660_610	U (7)	−	4.80	CC3	2015	Bradi1g74916.1	1:71954350-71958951
Contig49789_1300	U (4)	−	4.58	CC3	2013	Bradi4g37410.1	4:42546079-42548805
Contig34464_527	U	−	6.241	CC1	2015	Bradi2g61830.1	2:58209279-58216738
Contig34464_527	U	−	5.755	CC1	2013	Bradi2g61830.1	2:58209279-58216738
Contig49750_636	U		6.027	CC3	2015	Bradi2g62550.1	2:58209279-58216738

*U, unmapped marker. Where a marker was unmapped its predicted LG, based on the most significant BLAST alignment to the B. distachyon genome and position of this alignment to the Lolium perenne linkage map of Pfeifer et al. (2013), is recorded in brackets. If the predicted LG from the BLAST alignment differs from the linkage mapping derived LG, this is also recorded in brackets*.

On LG 1, significantly associated markers were found between map positions 0.0 and 29.8 cM, with the maximum –log*P* value of 11.99 in 2013 (marker contig42271_467 at 14.5 cM) and 12.04 in 2015 (marker contig6965_1844 at 28.7 cM). A further unmapped marker (Contig7723_139), that, through BLAST alignment with the Brachypodium genome sequence and reference to the perennial ryegrass GenomeZipper of Pfeifer et al. (2013), could be predicted to be located on LG 1, achieved a –log*P* value of 14.81 in 2013. On LG 2, significant marker associations were found over a smaller mapping distance between 42.7 and 62.8 cM. The maximum –log*P* values obtained were 8.90 in 2013 and 10.39 in 2015 (in both cases for marker Contig36905_912) at position 53.8 cM.

As the vast majority of significant marker associations were found on LGs 1 and 2, and *S* and *Z* are located on these LGs, these regions merited further investigation. The relative positions of significantly associated markers and the two *DUF247* candidate genes for *S* and *Z* was investigated using a comparative genomics approach where the physical positions of candidate gene sequences and marker sequences were determined in Brachypodium by BLAST alignment (Table 1). This enabled additional markers that were unmapped in perennial ryegrass to be located relative to the *S* and *Z DUF247* candidate gene homologues on the Brachypodium physical assembly.

All significant markers that mapped to LG 1 aligned to genes on either Brachypodium chromosome two or three that, according to Pfeifer et al. (2013), share synteny with genes on LG 1 of perennial ryegrass. For the *S DUF247* homologue (Bradi2g35750), the closest significantly associated SNP, contig32050_681 (−log*P* = 9.23 in 2013) aligned to Bradi2g35740. This gene is adjacent to the *S DUF247* homologue 12.0 kb distant. This marker was unmapped in perennial ryegrass but another marker (contig42271_467) with a –log*P* score of 11.99 (2013), at 14.5 cM, aligned to Bradi2g35050 in the same locality. On the other *S DUF247* flank, SNP markers contig35923_413 at 5.2 cM produced a –log*P* score of 4.26 (2013) and contig40661_72 at 18.4 cM produced a –log*P* score 5.19. There were also several significant associations for markers that aligned to Brachypodium chromosome 3. Markers contig7723_139 (−log*P* = 14.81, unmapped in perennial ryegrass) and contig6965_1844 (−log*P* = 12.04) at 28.7 cM aligned to Bradi3g30810 and Bradi3g30670, respectively and two markers on contig41583 (−log*P* = 8.61) at 26.0 cM and marker contig7422_2494 (log*P* = 7.15) at 28.1 cM aligned to Bradi2g25870 and Bradi2g25745, respectively.

All significant markers that mapped to LG 2 aligned to genes on Brachypodium chromosome 5. For the *Z DUF247* homologue (Bradi5g23930), the SNP marker contig36905_912 that produced the highest –log*P* scores in both years (8.90 and 10.39 in 2013 and 2015, respectively) on LG 2 aligned to Bradi5g24040 and was 72.0 kb distant with only nine genes between. On the other *Z DUF247* flank, SNP markers associated with contig31123 produced –log*P* scores of 7.35 (2013) and 7.82 (2015). This marker aligns to Bradi23890, 36.9 kb separated by only three genes.

## Discussion

We examined the simplest known example of a multi-locus SI system, the grass system, which is known to be controlled gametophytically by at least two unlinked complementary loci. Performed over 2 years, the proportion of compatible to incompatible pollen grains of crosses between 52 related plants from a commercial plant breeding programme was evaluated. A novel approach was used where a complex pattern of pollination scores was distilled from a diallel cross of a perennial ryegrass population into principal components for which we coined the phrase “Compatibility Components”. These components were then subjected to GWAS. From these data, highly significant marker associations with CC scores were identified on LGs 1 and 2 that, through comparative genomics with Brachypodium, were found to be linked to previously described candidate genes for the S and Z loci, which are the major determinants of SI in grasses. Most striking of these results is the ability to identify (with reasonably high precision) known SI loci with few plants. This finding demonstrates the robustness of the GWAS approach to identifying SI loci in multi-locus systems. The method, using genetically diverse populations, may therefore be used to evaluate, and determine causal loci for, observations that do not fit with established genetic models. We have shown the veracity of this method in perennial ryegrass but propose that it is also amenable to other gametophytic SI systems controlled by unidentified multiple loci which are known to be expressed in a wide range of flowering plant families including Chenopodiaceae (Lundqvist et al., 1973), Ranunculaceae (Lundqvist et al., 1973; Lundqvist, 1990), Liliaceae (Lundqvist, 1991), and Fabaceae (Lundqvist, 1993).

The pollinations of the diallel cross showed distinctive yet complex patterns of family relationships from which no more than generalised conclusions on the genetics of SI could be inferred. SI within half-sibs was, as expected, greater than between half-sibs, as they would be expected to possess one of two alleles from each incompatibility locus of the common mother plant. The whole population shared a common pollen cloud derived from the around 400 parental plants of the previous generation so a degree of cross-incompatibility between half-sib families was also expected, even if the mother plants did not share common incompatibility alleles. In an extreme case, there is evidence that the maternal parents of half-sib family 1 and 2 probably have few, if any, incompatibility alleles in common as nearly all progeny crosses between these individuals of these two families were fully compatible. Observations suggest that the maternal parents of half-sib families 1, 3, and 4 share common incompatibility alleles.

Without an existing genetic model for SI, it would be extremely difficult to model incompatibility beyond these general observations. It even proved impossible to fit the pollination data to the accepted two locus gametophytic SI model. Each individual plant produced a unique set of pollination reactions with other members of the population. Two observations inconsistent with the normal operation of the two-locus SI system were self-pollen tube growth and reciprocal crosses that were fully compatible in one direction but not the other. Furthermore, this second inconsistency was not always repeated between years. Although we cannot rule out the possibility of misinterpretation of pollen tube growth observations, the perennial ryegrass SI system is imperfect. It is well known that self-seeding is common (Jenkin, 1931) and enhanced by high temperatures (Wilkins and Thorogood, 1992), which, in rye was inferred to be under genetic control (Gertz and Wricke, 1991). Furthermore, there are example SI studies in self-incompatible ryegrass species where it was impossible to fit a two-locus model to the results obtained from pollinations of F1 plants derived from single pair-crosses (McCraw and Spoor, 1983a,b). The researchers observed self-compatible plants and more than 16 incompatibility groups and were obliged to conclude that at least three loci were involved in the SI response.

This paper reports on a PCA procedure used to distil variation in pollination behaviour down to four CCs accounting for an accumulated total of 70% (2013) and 75% (2015) of the variance observed. CC loadings for each individual plant were used in a subsequent GWAS analysis. We were able to demonstrate the robustness of these scores for confirming the positions of the *S* and *Z* genetic loci known to be involved in the SI response of grasses with markers generating very high –log*P* significant association scores. Markers within a relatively narrow recombination distance of 13.5 cM were associated with *Z* compared to 25.2 cM with *S* with peak values coinciding as close to candidate *S* and *Z* genes as the marker mapping density could reasonably have expected to achieve. Although CC1 appeared to identify several loci, remarkably, CC2 and CC3 exclusively identified loci definitively associated with *Z* and *S* locations, respectively (see later in Discussion). This observation alone demonstrates the power of the GWAS approach to identifying individual SI loci in multi-locus gametophytic systems.

Our results also make it tempting to speculate on other regions significantly associated with CC scores. This is especially so with associations with markers located on LG 3 and LG 6. A locus on LG 3 has been postulated to be involved with SI in perennial ryegrass through epistasis with the *S* locus causing certain *S* – LG 3 locus allele combinations to induce an incompatible reaction overriding the operation of the S-Z system (Thorogood et al., 2002). Two RFLP markers from this study, CDO920 and WG889, were the most closely linked with the causative gene on LG 3 and share sequence homology with Brachypodium genes Bradi2g41400 and 2g46140, respectively. These genes map to the perennial ryegrass GenomeZipper (Pfeifer et al., 2013) at distances of 37.2 and 40.1–40.3 cM. The significantly associated markers on LG 3 revealed by the GWAS coincide with Brachypodium genes Bradi2g43650 and 2g61010 that map to the GenomeZipper at distances of 40.9–41.3 and 60.5–61.3 cM. The first of these, at least, coincides closely enough to the markers identified by Thorogood et al. (2002), less than one cM distant, to speculate that the locus identified in both populations is encoded by the same SI-associated gene. The involvement of loci on LG 3 and LG 6 has also been reported acting as self-fertility modifiers of the *S-Z* system in rye (Wehling et al., 1995). It is impossible to say definitively if these loci coincide with the positions of the significantly associated markers revealed in the current study, as the authors did not provide any marker data for these locations. Furthermore, the levels of significance attached to the GWAS associations are far lower than the associations found for LG 1 and LG 2 markers and must be regarded with reservation. These additional associations do however deserve further investigation, and aberrant genotypes with pollination scores conflicting with S-Z model predictions could be selected as sires for future mapping families.

In addition to the loci revealed in this GWAS, a mapped locus (or loci) on LG 5, for which the only variant that has been revealed is one for self-fertility, has been identified in perennial ryegrass (Thorogood et al., 2005; Arias Aguirre et al., 2013) and in rye (Fuong et al., 1993).

The distribution of LG 1 linked markers significantly associated with compatibility is worthy of further discussion. In the current absence of a contiguous perennial ryegrass physical chromosome assembly, comparative genomics enabled reasonable estimates of physical distances of genes or loci to be made. The Brachypodium genome sequence provided a useful tool for comparative genomics studies in other grasses. The shared synteny of the perennial ryegrass with Brachypodium (and other model grass species) has been determined (Jones et al., 2002; Pfeifer et al., 2013). Perennial ryegrass LG 1 shares synteny with two blocks of Brachypodium chromosome 2 flanking a central block of chromosome 3. In our association studies, significant associations with CCs were found over a recombination distance of 25 cM on this LG that covers homoeologous regions on Brachypodium chromosomes 2 and 3 (Table 1) in general accordance with the shared synteny arrangement determined by Pfeifer et al. (2013). It would be straightforward to assume that all of these significantly associated markers are significant solely because of linkage to the *S* locus. However, markers aligned to genes on all three Brachypodium chromosome blocks and, although it is not possible to determine what physical distance this represents, it is clearly a very large proportion of the chromosome. Marker contig7723_139 in particular, aligning to Brachypodium Bradi3g30810 on chromosome 3 produced the highest -log*P* score (14.81) in the whole study. In terms of the perennial ryegrass LG 1 recombination distance and the Brachypodium physical distance, this marker is separated to such an extent that its significant association is suggestive of the influence of another incompatibility element linked to the *S* locus. However, this possibility has to be tempered by the fact that the physical distance is estimated in the *B. distachyon* homoeologous region, the gene order of which may have significantly diverged from that of perennial ryegrass.

The associations with the location of the *DUF247* candidate gene at *Z* are far simpler to interpret as all of the significant markers align to Brachypodium gene homologues located on Brachypodium chromosome 5, covering a physical distance of approximately 3 Mbp. There is a clear association with a region centred around the middle of the perennial ryegrass recombination map on LG 2, peaking around the Z *DUF247* gene, though this is likely to be a telomeric position as indicated by the predicted physical map locations of markers on homoeologous Brachypodium chromosome 5.

We have shown that the methodology presented here enables the identification of major SI loci, using a relatively small population size. The study does fall short of actually pinpointing the causal genes, and even though using an advanced (F13) population undoubtedly increased the resolution, by leveraging historical recombination events, linkage disequilibrium was still observed over several cM. Furthermore, the SNP marker density at considerably less than one SNP for each functional gene could not be reasonably expected to identify causal genes. However, our research has demonstrated that similar sized breeding populations (essentially restricted because of the number of pollinations required to create a complete dataset) subjected to a restrictive but extensive pedigree, with more saturated SNP coverage, could be used for SI studies in other crop plant species. For undomesticated species, populations subjected to restrictive within population cross-pollination over several generations in natural habitats could be used. We did not attempt to optimise population and marker parameters in this current grass study but simply worked with what was available in our chosen species of study. Ultimately, as with any GWAS study, the success and accuracy of SI locus or gene discovery will be determined by marker density and the number of historical recombinant events experienced by the population under investigation. Despite the limitations encountered, we have been able to demonstrate the feasibility of a new method for studying the hitherto recalcitrant nature of the genetics of complex multi-locus SI systems in flowering plants. Moreover, evidence indicated that the methodology, by exploiting information obtained from a diverse population panel, also has the potential to uncover the involvement of additional loci in existing model SI systems.

From a practical viewpoint, information on the number and location of SI loci is important when developing strategies for using restricted cross-incompatibility in parental populations for F1 hybrid population construction based on SI locus genotype predictions. Such schemes have been advanced based on genotype selection using a two-locus model (England, 1974; Pembleton et al., 2015) but could be compromised by the involvement of additional compatibility loci.

## Materials and methods

### Plant materials and semi-*in-vivo* pollinations

The plant population used in this study was obtained after 12 generations of half-sib family selection from a base of seven plants of diverse origin. Three plants were ecotypes originating from Northern Italy, two were from the variety “Melle Pasture” and two were Ryegrass Mosaic Virus resistant survivors selected from the cultivar, “Aberystwyth S23”. Two further plants were added at the tenth and eleventh generations. These plants derived from genotypes of the cultivars “Jumbo” and “Twystar,” respectively that had been top-crossed using seven genotypes of the cultivar “AberDart.” The thirteenth generation was derived from a poly-cross between 415 plants. Half-sib progeny seed was harvested separately from each of the 415 plants and 96 progeny families were selected for progeny testing. Four of the 400 families were then selected based on progeny plot trials for agronomic traits as the basis for further breeding: Remnant half-sib progeny seeds from four mother plants were used to create the thirteenth generation consisting of 55 out of 240 selected individuals, only 52 of which survived and are included in this current study. Thus, each of the four half-sib families consisted of individuals with a common maternal parent pollinated by an unidentified paternal individual from the 415 twelfth generation plants.

Three clonal replicates of vegetatively maintained plants were grown in 15 cm diameter pots of John Innes No. 3 (John Innes Manufacturers Association, Reading, UK) compost in a frost-free glasshouse and were re-potted each year. Plants flowered after a natural vernalisation period of short days and low temperatures, in response to naturally lengthening days. Semi*-in-vivo* pollinations and subsequent slide preparations of pollinated stigmas were made according to Thorogood et al. (2002).

In 2013, diallel cross-pollinations between 50 plants (10, 11, 14, and 15 plants from the four half-sib families) were made. All plants were self- and cross-pollinated and 1,969 pollinations were classified from a possible total of 2,500. In 2015, an extra plant was added to both half-sib families one and two and all plants were again self- and cross-pollinated. A total of 2,496 pollinations were classified from a possible total of 2,704. Not all crosses were possible due to flowering time differences and the feasibility of carrying out such a large number of cross-pollinations in a short flowering period. Additionally, some pollinations were excluded from the analysis, as they could not be scored because pollen grains were inviable and failed to illicit any fluorochrome response to the aniline blue stain. Repeat pollinations of poorly presented slides were made whenever possible. We attempted to score pollinations according to proportions expected assuming a two locus model, i.e., fully incompatible, half-compatible, three-quarters compatible and fully compatible. However, for many pollinations, it was difficult to assign these compatibility scores with any certainty so we scored plants subjectively on a 0–10 scale as follows: 0 = Fully incompatible, 1–2 = Largely incompatible with a small proportion of compatible grains, 3 = Less than half-compatible, 4 = Half-compatible, 5 = More than half-compatible, 6 = Less than three-quarters compatible, 7 = Three-quarters compatible, 8 = More than three-quarters compatible but considerably less than fully compatible, 9 = Very close to fully compatible but with one or two incompatible grains observed, 10 = Fully compatible.

### Self-incompatibility 50 index

To estimate the degree of cross compatibility, a statistical model to describe the overall pairwise crosses between or within families was developed. The distribution of all pairwise crosses did not fit a normal distribution. Therefore, many routinely used statistical metrics or tests, such as ANOVA, are not applicable. To overcome this, we realised, when plotting the quantiles of cross compatibility scores the resulting plot resembled a classic sigmoid shaped curve (Supplementary Figures 1, 2). Using a self-starting four-parameter logistic shown in Equation (1) the mid-point of the inflection (*D*) of the curve between the lower (*A*) and upper asymptotes (*B*) of the quantiles (*x*) of the cross score (*Y*) could be determined. The equation also incorporates a numerical scaling parameter (C). The inflection point *D* was defined as the point where 50% of the pollinations were self-incompatible (SI_50_), which is akin to the lethal dose 50 (LD_50_) measure commonly used in toxicology studies. The SI_50_ was estimated using non-linear (weighted) least squares (nls) with the self-start four-parameter logistic model (SSfpl) functions in R. To ensure the asymptotes were correctly assigned, values of −100:−1 and 101:200 were added to the data with cross scores of 0 and 10, respectively. Additionally, all cross score values were incremented by one, as values of zero are incompatible with this model. To estimate the *R*^2^, the sum of the residuals (*r*) from the model (of the data, excluding the artificial values added) squared was divided by the squared valued of each cross score (*x*) minus the mean ($\overset{®}{x}$) cross score of the population. The resulting division was subtracted from one as shown in Equation (2).

Eq.1 Y=A × (B − A1+e(D−xc))

$$\begin{array}{l} Eq.2\;R^{2}=1-\;\frac{\sum^{\;}\left(r^{2}\right)}{{{\sum \left(x-\bar{x}\right)}^{\;}}^{2}} \end{array}$$

The R workflow for the modelling is described by Yates (2017).

### Plant genotyping

Plants from the breeding population were genotyped using a custom Illumina iSelect SNP genotyping array developed from Next Generation Sequencing outputs described in Blackmore et al. (2015). Results of the genotyping are fully described in Grinberg et al. (2016) but briefly, of the 3,775 markers on the array, 2,764 were surveyed in the breeding population and a total of 2,461 and 2,464 markers were suitable after QC filtering for genome wide association analysis in the 2013 and 2015 sets, respectively. All marker sequences cited in this manuscript are available through the supplementary data of Blackmore et al. (2015) and SNP genotype calls are available on demand.

### Pollination diallel analysis

The incompatibility relationships between the plants were evaluated separately for 2013 and 2015 pollinations. The diallel compatibility matrix was converted into a similarity matrix using Euclidean distance where missing values were removed in the estimation of similarity between genotypes. To reduce the complexity further, a PCA was used on the Euclidean distance based similarity matrix. In so doing, the two-dimensional This was done without scaling, using singular value decomposition of the similarity matrix. Thus, the PCs relate to a similarity of pollination behaviour, in an enclosed, perfectly panmictic population. Clustering based on genotype PCs was made using hierarchical clustering of the Euclidean distance (Ward, 1963) of the first four PCs. All statistical analyses were made in the R statistical environment (version: 3.2.2, R Core Team, 2015). Graphical representations of the results were created using “ggplot2” package (version: 2.1.0, Wickham, 2009) with the exception of the heat maps of the diallel of pollinations which were created using the “gplots” package (version: 2.17.0) and the 3D scatterplots were rendered using the “scatterplot3d” package.

### Genome wide association analysis

The first four PC scores obtained from the analysis of pollination scores were used to describe relative plant compatibility phenotypes and were subjected to an association analysis with segregating genome-wide markers. Subsequent to PCA, the PCs were described as “compatibility components” (CCs) for reasons explained in the results section. Calculations were performed using the software package GenStat 17th edition. In GenStat, the “QTL analysis” module and the “Single trait association analysis” function was used. Although population structure was likely to be minimal (see Grinberg et al., 2016), structure was accounted for using PCA (eigenanalysis). Significant associations were determined by calculation of a Bonferroni correction threshold value for multiple significance-test correction. Although 2,461 and 2,464 markers were input for the analysis for the 2013 and 2015 datasets, respectively, 200 markers with a minor allele frequency <0.05 were excluded from the final analysis leaving, a total of 2,261 and 2,264 segregating loci for evaluations performed in 2013 and 2015, respectively.

### Marker mapping

A genetic recombination map was used as a reference for initially anchoring genomic regions showing significant association with stigma-pollen self- and cross-incompatibility. The map was integrated using Joinmap 4.1 (Van Ooijen, 2006) from three unrelated, mapping families, two perennial ryegrass bi-parental families and an F2 family that had all been mapped with segregating markers from the *Lolium* customised Illumina SNP array of Blackmore et al. (2015). Seven LGs were separated using an independence LOD score of up to 8.0. Further unmapped markers were included in the subsequent association analysis. A summary of map coverage is given in Supplementary Table 3.

In the current absence of a contiguous perennial ryegrass genome sequence assembly for each chromosome, all markers (mapped and unmapped) were BLASTN (version: 2.2.28, Altschul et al., 1990) searched against a Brachypodium genome database (version 1.0.31, downloaded from plants.ensembl.org). Comparisons of the relative physical positions of SNP polymorphisms with annotated Brachypodium genes including homologues of candidate *S* and *Z* genes was then made. The BLAST results were filtered with a minimum *E*-value greater than 1e^−5^ and only the best match was retained, based on *E*-value. The positions of the markers were then aligned to the Brachypodium physical sequence based on the left-most position of the resulting alignment. Based on syntenic relationships between perennial ryegrass and Brachypodium, this enabled unmapped markers to be aligned to the Brachypodium genome positions and provided a check for the LG allocations determined by the integrated *Lolium* map.

All perennial ryegrass NGS sequence data can be accessed via Blackmore et al. (2015) and SNP genotype data from all individual plants is available on request.

## Author contributions

DT, BS, SY, and CM conceived the original research approach and designed the experiments; DT performed the *in-vitro* pollinations; MH and TB developed the Lolium SNP chip array; DT, MH and TB produced genetic maps; data analyses were made by DT, SY and LS and the article was written and co-ordinated by DT with contributions from all authors.

### Conflict of interest statement

The authors declare that the research was conducted in the absence of any commercial or financial relationships that could be construed as a potential conflict of interest.
