# Supplementary material for: A Novel Multivariate Approach to Phenotyping and Association Mapping of Multi-Locus Gametophytic Self-Incompatibility Reveals S, Z, and Other Loci in a Perennial Ryegrass (Poaceae) Population
Source: Front Plant Sci. 2017 Aug 2;8:1331. doi: 10.3389/fpls.2017.01331 (PMC5539123; doi:10.3389/fpls.2017.01331)
Supplement: Supplementary file 1 [file DataSheet1.DOCX]

Supplementary Material

**A novel multivariate approach to phenotyping and association mapping of multi-locus gametophytic self-incompatibility reveals *S*, *Z* and other loci in a perennial ryegrass (Poaceae) population**

**Daniel Thorogood^1*^, Steven Yates^2^, Chloé Manzanares^2^, Leif Skot^1^, Matthew Hegarty^1^, Tina Blackmore^1,4^, Susanne Barth^3^, Bruno Studer^2^**

**Correspondence: Corresponding author:** [dnt@aber.ac.uk](mailto:dnt@aber.ac.uk)

# Supplementary Data

# Supplementary Figures and Tables

Supplementary Table 1. Correlation coefficients (*R*) of within and between half-sib family pollinations made in 2013 and 2015.

|  |  | ♂ | | | | |
| --- | --- | --- | --- | --- | --- | --- |
|  |  | **h-sib 1** | **h-sib 2** | **h-sib 3** | **h-sib 4** |  |
| ♀ | **h-sib 1** | 0.60 | 0.42 | 0.74 | 0.52 | 0.67 |
|  | **h-sib 2** | 0.43 | 0.64 | 0.54 | 0.63 | 0.69 |
|  | **h-sib 3** | 0.63 | 0.65 | 0.83 | 0.63 | 0.73 |
|  | **h-sib 4** | 0.55 | 0.55 | 0.61 | 0.71 | 0.64 |
|  |  | 0.63 | 0.70 | 0.72 | 0.61 | 0.68 |

All correlations *P* < 0.001 (degrees of freedom ranging between 98 and 223)

Supplementary Table 2. Correlation coefficients (*R*) pollinations made of each genotype as female or male parent with other members of the population.

| **Genotype** | **♀** | **df** | **P** | **♂** | **df** | ***P*** |  | **Genotype** | **♀** | **df** | **P** | **♂** | **df** | ***P*** |
| --- | --- | --- | --- | --- | --- | --- | --- | --- | --- | --- | --- | --- | --- | --- |
| 5 | 0.80 | 40 | <.001 | 0.53 | 48 | <.001 |  | 222 | 0.82 | 41 | <.001 | 0.68 | 36 | <.001 |
| 12 | 0.66 | 37 | <.001 | 0.96 | 48 | <.001 |  | 223 | 0.74 | 35 | <.001 | 0.56 | 49 | <.001 |
| 27 | 0.57 | 33 | <.001 | 0.58 | 34 | <.001 |  | 228 | 0.92 | 40 | <.001 | 0.66 | 44 | <.001 |
| 33 | 0.45 | 41 | <.01 | 0.69 | 22 | <.001 |  | 231 | 0.67 | 36 | <.001 | 0.71 | 14 | <.01 |
| 39 | 0.62 | 36 | <.001 | 0.67 | 47 | <.001 |  | 233 | 0.67 | 31 | <.001 | 0.89 | 24 | <.001 |
| 42 | 0.74 | 41 | <.001 | 0.41 | 38 | <.05 |  | 235 | 0.95 | 36 | <.001 | 0.89 | 46 | <.001 |
| 43 | 0.72 | 33 | <.001 | 0.30 | 43 | <.05 |  | 238 | 0.58 | 32 | <.001 | 0.50 | 45 | <.001 |
| 45 | 0.70 | 35 | <.001 | 0.86 | 32 | <.001 |  | 294 | 0.60 | 27 | <.001 | 0.79 | 13 | <.01 |
| 46 | 0.78 | 45 | <.001 | 0.67 | 34 | <.001 |  | 295 | 0.48 | 35 | <.01 | 0.72 | 44 | <.001 |
| 47 | 0.46 | 38 | <.01 | 0.62 | 12 | <.05 |  | 298 | 0.81 | 34 | <.001 | 0.48 | 46 | <.001 |
| 115 | 0.85 | 41 | <.001 | 0.79 | 42 | <.001 |  | 301 | 0.63 | 38 | <.001 | 0.88 | 33 | <.001 |
| 123 | 0.65 | 36 | <.001 | 0.57 | 38 | <.001 |  | 307 | 0.67 | 41 | <.001 | 0.75 | 33 | <.001 |
| 131 | 0.89 | 30 | <.001 | 0.63 | 34 | <.001 |  | 314 | 0.60 | 42 | <.001 | 0.63 | 30 | <.001 |
| 132 | 0.79 | 43 | <.001 | 0.89 | 48 | <.001 |  | 318 | 0.69 | 37 | <.001 | 0.58 | 18 | <.05 |
| 133 | 0.67 | 40 | <.001 | 0.61 | 49 | <.001 |  | 320 | 0.37 | 40 | <.05 | 0.56 | 45 | <.001 |
| 134 | 0.84 | 36 | <.001 | 0.42 | 33 | <.01 |  | 323 | 0.00 | 27 | NS | 0.91 | 41 | <.001 |
| 136 | 0.50 | 35 | <.01 | 0.76 | 34 | <.001 |  | 329 | 0.83 | 40 | <.001 | 0.71 | 47 | <.001 |
| 138 | 0.69 | 45 | <.001 | 0.90 | 46 | <.001 |  | 331 | 0.90 | 39 | <.001 | 0.69 | 42 | <.001 |
| 143 | 0.64 | 27 | <.001 | 0.70 | 48 | <.001 |  | 332 | 0.80 | 37 | <.001 | 0.69 | 46 | <.001 |
| 145 | 0.53 | 42 | <.001 | 0.83 | 27 | <.001 |  | 334 | 0.45 | 26 | <.05 | 0.15 | 15 | NS |
| 148 | 0.56 | 46 | <.001 | 0.51 | 46 | <.001 |  | 337 | 0.53 | 34 | <.001 | 0.62 | 34 | <.001 |
| 204 | 0.96 | 35 | <.001 | 0.79 | 31 | <.001 |  | 342 | 0.66 | 31 | <.001 | 0.70 | 18 | <.01 |
| 213 | 0.78 | 38 | <.001 | 0.82 | 45 | <.001 |  | 345 | 0.82 | 31 | <.001 | 0.58 | 39 | <.001 |
| 216 | 0.69 | 43 | <.001 | 0.93 | 34 | <.001 |  | 347 | 0.82 | 40 | <.001 | 0.68 | 41 | <.001 |
| 218 | 0.66 | 34 | <.001 | 0.84 | 32 | <.001 |  | 348 | 0.74 | 30 | <.001 | 0.73 | 40 | <.001 |

Supplementary Table 3 Summary statistics for numbers and density of SNP markers mapped using the integrated map of Blackmore et al. 2015).

| Linkage group | Length (cM) | Number of markers | Mapped marker density (n/cM) | Number of markers | Mapped marker density (n/cM) |
| --- | --- | --- | --- | --- | --- |
|  |  | 2013 | | 2015 | |
| 1 | 87.6 | 236 | 2.69 | 237 | 2.71 |
| 2 | 127.0 | 251 | 1.98 | 251 | 1.98 |
| 3 | 99.2 | 257 | 2.59 | 257 | 2.59 |
| 4 | 131.2 | 330 | 2.52 | 331 | 2.52 |
| 5 | 89.2 | 153 | 1.72 | 153 | 1.72 |
| 6 | 114.0 | 267 | 2.34 | 267 | 2.34 |
| 7 | 106.0 | 278 | 2.62 | 278 | 2.62 |
| Total mapped | 754.2 | 1772 | 2.35 | 1774 | 2.35 |
| Unmapped |  | 689 | | 690 | |
| Total mapped and unmapped |  | 2461 | | 2464 | |

## Supplementary Figures


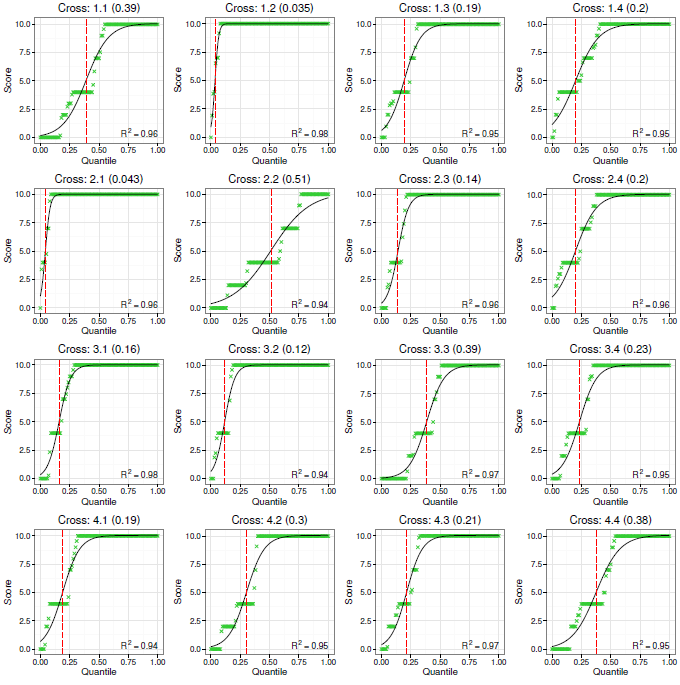


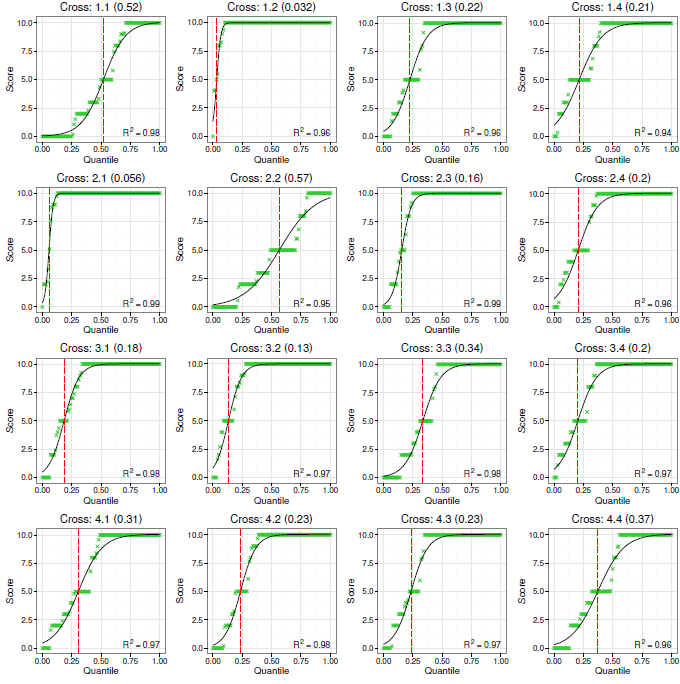


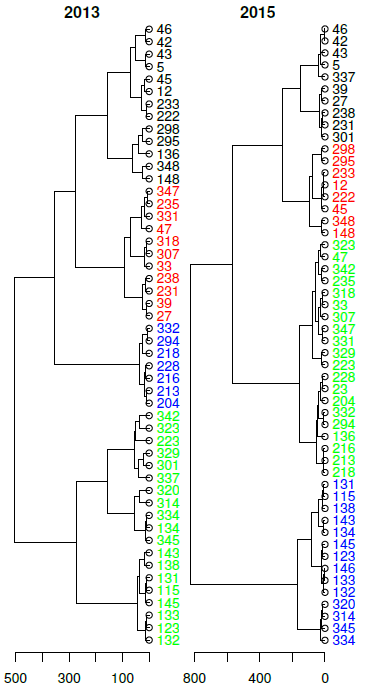


**
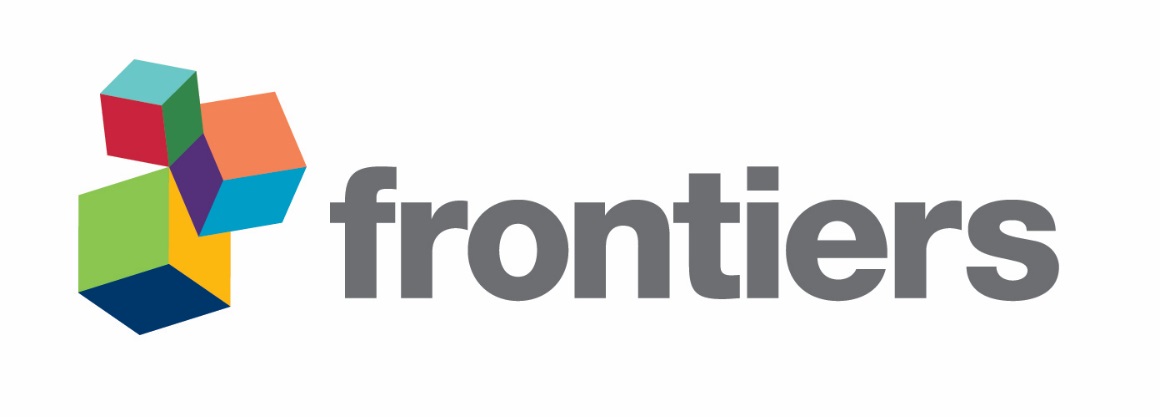
**

**Supplementary Figure 1.** 2013 cross-compatibility quantiles within and between half-sib families. Compatibility score (1-10, y-axis) is plotted against quantile (every 0.01, x-axis). Cross numbers in each graph title are given as two numbers (x.y), the first designating the maternal half-sib family and the second, the paternal half-sib family and is then followed by the SI_50_ score in brackets. The quantiles are shown as green crosses and the predictions of the modelling as a solid black line. The SI_50_ is shown as a vertical red dashed line. R^2^ for the model is shown in the lower right corner of each graph.

**Supplementary Figure 2.** 2015 cross-compatibility quantiles within and between half-sib families. Compatibility score (1-10, y-axis) is plotted against quantile (every 0.01, x-axis). Cross numbers in each graph title are given as two numbers (x.y), the first designating the maternal half-sib family and the second, the paternal half-sib family and is then followed by the SI_50_ score in brackets. The quantiles are shown as green crosses and the predictions of the modelling as a solid black line. The SI_50_ is shown as a vertical red dashed line. R^2^ for the model is shown in the lower right corner of each graph.

**Supplementary Figure 3.** Dendrograms showing principal components for 2013 and 2015 of cross-compatibility scores. The genotype numbers (5-47 = half-sib family 1; 115-148 = half-sib family 2; 204-298 = half-sib family 3; 301-348 = half-sib family 4) are coloured based on hierarchical clustering into four groups using the Ward method.
